# Attenuated DNA damage responses and increased apoptosis characterize human hematopoietic stem cells exposed to irradiation

**DOI:** 10.1038/s41598-018-24440-w

**Published:** 2018-04-17

**Authors:** Shahar Biechonski, Leonid Olender, Adi Zipin-Roitman, Muhammad Yassin, Nasma Aqaqe, Victoria Marcu-Malina, Melanie Rall-Scharpf, Magan Trottier, M. Stephen Meyn, Lisa Wiesmüller, Katia Beider, Yael Raz, Dan Grisaru, Arnon Nagler, Michael Milyavsky

**Affiliations:** 1 Department of Pathology, Tel-Aviv University, Tel-Aviv, 69978 Israel; 2 Sackler Faculty of Medicine, Tel-Aviv University, Tel-Aviv, 69978 Israel; 3 Cytogenetic Unit, Laboratory of Hematology, Chaim Sheba Medical Center, Tel-Hashomer, Israel; 4 Department of Obstetrics and Gynecology, Gynecological Oncology, University of Ulm, Prittwitzstrasse 43, Ulm, Baden-Wuerttemberg, 89075 Germany; 5 Department of Molecular Genetics, University of Toronto, ON M5G, 1L7 Canada; 6 Clinical Genetics Service, Memorial Sloan Kettering Cancer Center, New York, New York, USA; 7 Center for Human Genomics and Precision Medicine, School of Medicine and Public Health, University of Wisconsin, Madison, Wisconsin USA; 8 Hematology Division, Chaim Sheba Medical Center, Tel-Hashomer, Israel; 9 Department of Obstetrics and Gynecology, Gynecologic Oncology Division, Lis Maternity Hospital, Tel Aviv Sourasky Medical Center, Tel-Aviv, 64239 Israel

## Abstract

Failure to precisely repair DNA damage in self-renewing Hematopoietic Stem and early Progenitor Cells (HSPCs) can disrupt normal hematopoiesis and promote leukemogenesis. Although HSPCs are widely considered a target of ionizing radiation (IR)-induced hematopoietic injury, definitive data regarding cell death, DNA repair, and genomic stability in these rare quiescent cells are scarce. We found that irradiated HSPCs, but not lineage-committed progenitors (CPs), undergo rapid ATM-dependent apoptosis, which is suppressed upon interaction with bone-marrow stroma cells. Using DNA repair reporters to quantify mutagenic Non-Homologous End Joining (NHEJ) processes, we found that HSPCs exhibit reduced NHEJ activities in comparison with CPs. HSPC-stroma interactions did not affect the NHEJ capacity of HSPCs, emphasizing its cell autonomous regulation. We noted diminished expression of multiple double strand break (DSB) repair transcripts along with more persistent 53BP1 foci in irradiated HSPCs in comparison with CPs, which can account for low NHEJ activity and its distinct control in HSPCs. Finally, we documented clonal chromosomal aberrations in 10% of IR-surviving HSPCs. Taken together, our results revealed potential mechanisms contributing to the inherent susceptibility of human HSPC to the cytotoxic and mutagenic effects of DNA damage.

## Introduction

Life-long blood production depends on HSPCs - a subset of primitive hematopoietic cells endowed with high self-renewal potential. HSPCs give rise to CPs with limited or no self-renewal, which in turn, differentiate into various mature blood cells. Analysis of human HSPC isolated from newborn, young, and elderly individuals by DNA sequencing has revealed that HSPCs serve as a reservoir for genetic changes, including mutations in genes implicated in leukemia; thus, they are a likely cell of origin for hematopoietic malignancies^1–5^. DNA replication and cellular metabolism are among the endogenous sources of DNA damage that can contribute to mutagenesis and carcinogenesis. However, exposing the body to exogenous inducers of DNA damage, such as IR and certain chemotherapeutic drugs can greatly increase the rate and occurrence of genomic aberrations. Thus, these inducers are implicated in the development of bone marrow failure, myelodysplastic syndrome as well as de novo and therapy-related leukemia^6,7^. DNA Double Strand Breaks (DSBs) are the most lethal and dangerous forms of DNA damage induced by IR, and when left unrepaired or misrepaired, they can lead to cell death or potentially oncogenic mutations^6,8^. To protect genome stability and integrity, multicellular organisms have developed highly sophisticated DNA-damage response (DDR) pathways that mediate and control DNA repair, cell-cycle checkpoints, and DNA damage-induced apoptosis. Activation and coordination of various DDR pathways occur after DSB formation by stimulating DDR kinases, including ATM, DNA-PK, and CHK2 as well as their effectors such as p53 and NF-kB^9^.

DSB repair can occur via Non-Homologous End Joining (NHEJ) or Homologous Recombination (HR) pathways that differ in their intrinsic mutagenicity, regulation, and molecular machineries. Canonical NHEJ can join DSBs without the need for homology; it is considered partially error-free and operates in all cell cycle stages. The Alternative EJ (Alt-EJ) pathway is a genetically distinct arm of NHEJ. It requires DSB end processing when searching for microhomologies, resulting in deletions of the sequences between the microhomology regions^6,7^. HR, in contrast with NHEJ, relies on an undamaged homologous template for DSB repair; it is considered error-free and is restricted to the S phase of the cell cycle^7^. Because HSPCs are largely quiescent during steady state, their DSBs are repaired via the canonical- or Alt- NHEJ pathways. Both the canonical NHEJ and Alt-EJ pathways have been implicated in the generation of genomic structural variants and chromosomal translocations in human cells and cancers^10–12^. Importantly, chromosomal translocations are the hallmarks of hematological malignancies and are considered an initiating transforming event^6^. When the amount or severity of DNA damage in HSPCs surmount its repair capacity, one of the pre-programmed pathways including apoptosis, precipitous differentiation, and senescence is activated^13,14^.

In recent years, several studies that characterized the response of murine HSPCs to IR revealed the preferential utilization of error-prone NHEJ and the increased resistance to IR-induced cell death than their respective progeny^15,16^. Conversely, the initial data sets on DDR in human HSPCs suggested that they have a delayed DSB rejoining capacity and increased IR-sensitivity, relative to CPs isolated from cord blood^17,18^. Collectively, these studies revealed potentially important distinctions in IR-induced DDR in human versus rodent HSPCs as well as between HSPCs and CPs, however, the underlying mechanism remains poorly understood.

In particular, very little is known regarding those factors that affect human HSPC survival after DSB induction as well as the activity and efficiency of the major DNA repair pathways, although the importance of intact DDRs in preventing leukemogenesis is well established. To bridge this gap, we analyzed in detail the IR-induced cell death and activity of NHEJ repair pathways in human HSPCs and CPs derived from cord blood and bone marrow. Importantly, we observed the rapid induction of caspase-dependent cell death after IR in HSPCs, specifically, that is positively regulated by ATM, but can be suppressed by interaction of HSPCs with bone marrow stroma. Moreover, we found that human HSPCs exhibited lower NHEJ pathway activity in comparison with CPs. Importantly, surviving HSPCs transmitted chromosomal aberrations to their progeny. Collectively, our study emphasizes the inherent vulnerability of human HSPCs to exogenous DNA damage with potential adverse consequences that include bone marrow failure and leukemogenesis.

## Materials and Methods

### HSPCs and CPs purification

Samples of cord blood (CB) were obtained according to procedures approved by the institutional review boards of the Sheba Medical Centre, Israel and the Tel Aviv University. Informed consent was obtained from all subjects. CD34^+^ CB cells were enriched by positive selection with MACS CD34 + ultra-pure kit (Miltheny) according to the manufacturer’s protocol. After enrichment, cells were nucleofected and incubated in StemSpan media (Stem Cell Technologies, Canada) supplemented with following human cytokines (Peprotech Asia): IL6(10ng/ml), SCF(50ng/ml), FLT3L(50ng/ml), G-CSF(10ng/ml), TPO(25ng/ml) and Penicillin/Streptomycin (P/S, 1%). Cells were maintained in a humidified incubator at 37 °C and 5% CO_2_ for 24 h. For sorting experiments, CD34+ samples were incubated with the following antibodies: CD45RA-FITC, CD38-PE-Cy7, CD34-APC, CD135-PE, CD90-biotin-Qdot605. Antibody details can be found in Supplementary Table 2. Cells were sorted on FACSAria II (BD Biosciences). Isotype controls as well as the forward-scatter and side-scatter properties of normal viable cord blood cells, were used to establish sorting gates.

### Hematopoietic CD34^+^ cells and stroma co-culture

OP9M2 murine stromal cells were kindly provided by Dr. H. Mikkola and were cultured in MEM-Alpha medium (Gibco, Life technologies), supplemented with FBS (20%), P/S (1%), L-glutamine (1%) as described elsewhere^19^. For co-culture with CD34^+^ cells, OP9M2 were plated in 24-well tissue culture treated plate, irradiated (20 Gy, Biobeam gamma-irradiator) and incubated in a stroma medium (see above) for 24 hours. Following the incubation, medium was aspirated, and CD34^+^-enriched cells were plated on stroma in StemSpan medium supplemented with cytokines (see above).

### Inhibitors

The following inhibitors were used: Z-vad FMK (Promega, 100 μM), Q-VD-OPh (Sigma, 20 μM), PV-1019 (Merck-Millipore,10 μM), CHK2 inhibitor II (Merck-Millipore,10 μM), NU-7441 (Adooq, 20 μM), KU60019 (Tocris, 10 μM). All inhibitors were added to cells prior to nucleofection or irradiation and maintained in the growth medium for the experiment duration.

### DNA repair assay

pimEJ5GFP and EJ2GFP-puro constructs were gift from Jeremy Stark (Addgene plasmids # 44026, 44025). pCMV-I-SceI expression plasmid was described elsewhere^20^. After CD34^+^ cell enrichment, cells were resuspended in nucleofection medium (P3 primary cell kit, Lonza) at 10^6^ cells/100 µl, including inhibitors as indicated. Plasmid were mixed at 1:1:1 ratio (repair plasmid: I-SceI expression plasmid: pBlue-script plasmid) and 10 µg of total DNA mixture was added to the nucleofection reaction. Cells were nucleofected with CD34^+^ program using Amaxa 4D nucleofector system (Lonza) and repair was normalized to the nucleofection efficiency of the same fraction using constitutively active EGFP construct. The proportion of EGFP expressing cells in HSPCs and CPs was determined in Sytox blue stain negative gate (indicative of viable cells) by flow cytometry after 24 h.

### Apoptosis analysis by flow cytometry

For cPARP analysis, cells were stained for surface markers, fixed in formaldehyde (1.4%, EMS), permeabilized in ethanol (100%) followed by labeling with anti-cPARP antibody (Invitrogen). Annexin V Apoptosis Detection Kit (BD PharMingen) and SYTOX Blue Dead Cell Stain (Invitrogen) were used according to manufacturer’s protocols.

**Figure 1:**
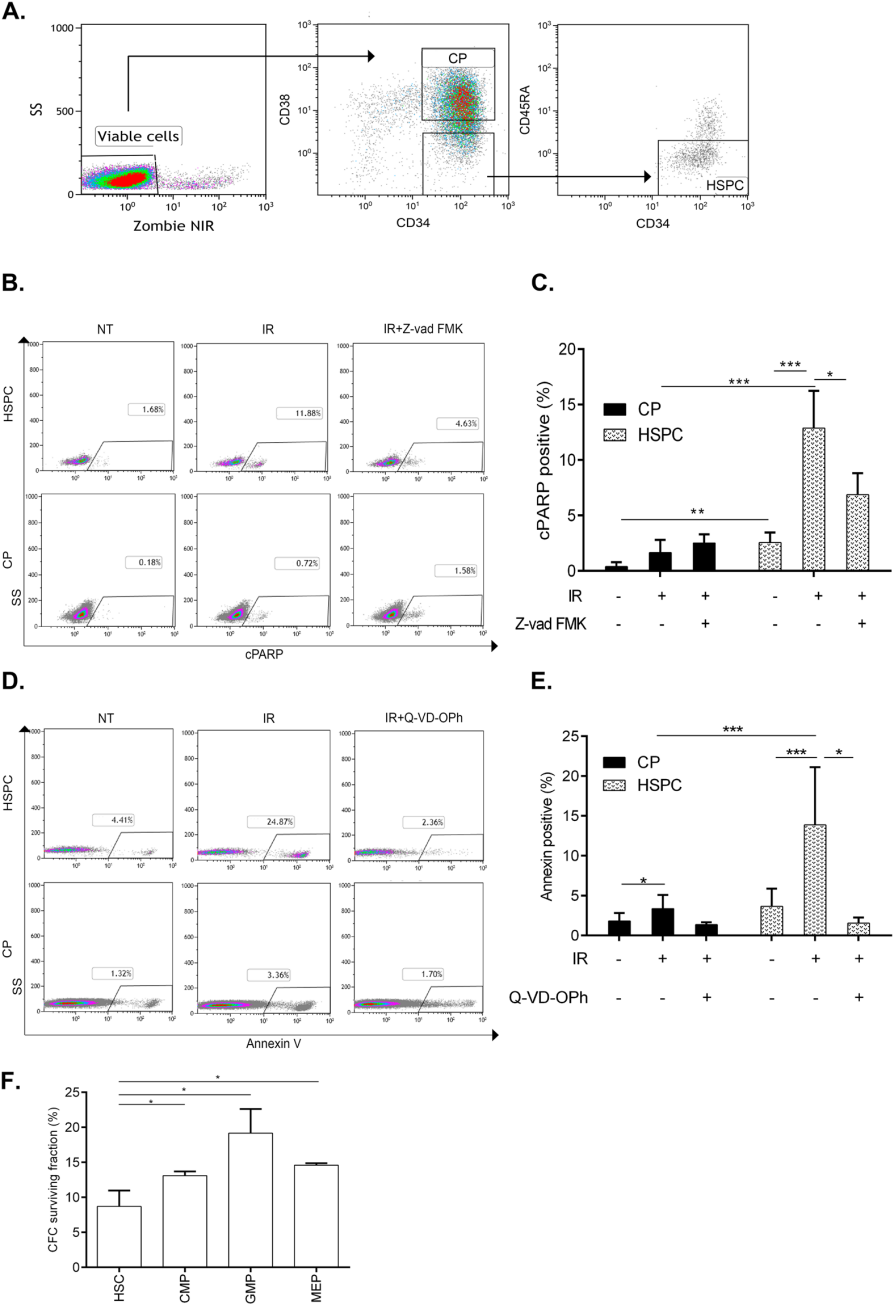
Analysis of IR-induced apoptosis in different fractions of primitive human hematopoietic cells. **(A)** Flow cytometry gating strategy for HSPCs and CPs. **(B,D)** Example of FACS data identifying cPARP^+^ **(B)** and AnnexinV^+^ **(D)** fractions in HSPCs and CPs gated as described in **(A)** 6 h after 3 Gy IR-treatment. **(C,E)** CD34^+^ cells were pre-treated with Z-vad-FMK (100 μM), Q-VD-OPh (20 μM) or DMSO for 1 h, irradiated with 3 Gy and analyzed for cPARP+ or Annexin V+ cells in HSPCs and CPs 6 h later, n ≥ 3. (**F**) Survival of clonogenic cells from sorted HSPC (CD34^+^CD38^−/low^CD90^+^45RA^−^), CMP (CD34^+^CD38^+^CD45RA^−^CD135^+^), GMP (CD34^+^CD38^+^CD45RA^+^CD135^+^) and MEP (CD34^+^CD38^+^CD45RA^−^CD135^−^) fractions following IR with 3 Gy. CFC surviving fraction was calculated by dividing number of colonies counted in the irradiated plates by the number of colonies from the same fraction scored in the non-irradiated plates, n = 3. Represented are mean values ± SD; *p < 0.05, **p < 0.01, ***p < 0.001.

Zombie NIR (Biolegend) staining was used to label dead cells prior to fixation. Cells were analyzed with Gallios (Beckman Coulter). Gating strategy is outlined in Fig. 1A. Briefly, cells with damaged membranes (defined by Zombie or SYTOX positivity) were gated out. Then, this “viable cell gate” was used to quantitate apoptosis markers in various subpopulations (Fig. 1B,D). Antibody details can be found in Supplementary Table 2.

### Quantitative Immunofluorescence

Sorted HSPCs and CPs were irradiated with 3 Gy (Biobeam 8000 Research Irradiator, Gamma service, UK). Cells were allowed to adhere to poly-Lysine coated slides (Electron Microscopy Sciences, PA) for 30 min at 37 °C prior to indicated time points. Cells were fixed with formaldehyde (2%)/Triton-X100 (0.2%), permeabilized in Triton (0.5%), followed by blocking (normal donkey serum) and labeled with antibodies against 53BP1 (1:3000 (Novus)), overnight at 4 °C. FITC-conjugated donkey anti-rabbit IgG was used as a secondary antibody. The number of foci was assessed using Image-J software.

### Karyotype Analysis

For the cytogenetic analysis of chromosomal aberrations, CD34+ cells were flow sorted into HSPC- and CP- fractions irradiated with 2 Gy or not and plated into methylcellulose (Methocult H4434, Stem Cell Technologies). Plates were incubated at 37 °C for 11–12 days. For metaphase preparation, Colcemid (0.2 µg/ml, Biological Industries, Israel) was added for 3 hrs followed by individual colonies isolation under microscope and processing for metaphase spreads as described elsewhere^21^. Colonies with >50 metaphases were karyotyped using G banding technique. Clonal aberration refers to the cases when identical chromosomal aberration was observed in at least three metaphases from the same colony.

### Bioinformatics analysis of DDR-related pathways

Expander tool kit^22^ was used to analyze the gene expression datasets detailed in Supplementary Table 3. Each dataset was normalized using Normalization algorithm provided by the Expander. Then, pre-defined gene sets were selected (Supplementary Table 4). For each subpopulation defined in the datasets, average expression of the gene set was calculated based on the expression values of the individual genes involved in the set. Statistical comparison was performed by Analysis of variance test (ANOVA) between the selected subpopulation compared to HSPCs set as a baseline.

### Statistical analysis

The significance of differences among groups was calculated via Student’s t-test and results were considered significant at P < 0.05. Each set of experiments was repeated at least three times on different dates. The data are presented as mean ± standard deviation (SD).

## Results

### Quiescent human HSPCs are exquisitely sensitive to IR-induced apoptosis compared with CPs

The hematopoietic system is hierarchically organized, whereby cells at various stages of differentiation can be distinguished by their cell surface molecules. Distinct DDR characteristics of murine HSPCs and various progenitor types have recently been addressed^23,24^, whereas a comparable analysis of human primitive hematopoietic subpopulations has lagged behind. In this study we decided to examine more closely the differences in the response to DSBs between two major subpopulations of primitive human hematopoietic cells: (1) CD34^+^CD38^low/−^CD45RA^−^ cells, which are highly enriched for quiescent primitive HSPCs, and (2) CD34^+^38^+^ cells that contain lineage-committed progenitor/precursor cells (Fig. 1A). We designated these fractions as “HSPCs” and “CPs”, respectively. To investigate the DDR and its outcomes in HSPCs and CPs, we irradiated CD34^+^ cord blood cells immediately after their isolation. IR-induced cell death was analyzed first as the most extreme DDR in HSPCs, overruling alternative responses such as DNA repair. To this end, we combined surface antigen staining with detection of specific apoptosis markers such as cleaved-PARP (cPARP) (Fig. 1B,C) and phosphatidylserine exposure on the cell surface, detected by AnnexinV binding (Fig. 1D,E). Using this strategy, we found that 12.9% of irradiated HSPCs became c-PARP positive 6 h post-IR, whereas only 1.6% of CPs exhibited PARP cleavage at this time point. Moreover, we found no further increase in the proporotion of cPARP^+^ HSPCs at the later time point (24 h post IR). Notably, HSPCs elicited higher levels of basal cPARP staining compared with CPs (Fig. 1B, C and Supplementary Figure 1). In agreement with PARP cleavage analysis, Annexin V based cell death quantitation revealed much higher rate of apoptosis in HSPCs realively to CPs (Fig. 1D,E). Pan-caspase inhibitors (Z-vad-FMK and Q-VD-OPh) significantly reduced the fraction of cPARP^+^ and AnnexinV^+^ HSPCs (Fig. 1B,D), further confirming the caspase-dependency of this rapid cell death process. Loss of clonogenic potential is a biological manifestation of cell death in HSPCs and CPs. Thus, we investigated clonogenic survival of HSPCs and various CPs sub-types (common cyeloid progenitor (CMP), granulocyte macrophage progenitor (GMP) and megakaryocyte–erythroid progenitor (MEP) exposed to 3 Gy of IR after isolation by flow sorting from cord blood. As can be seen in Fig. 1F, irradiated HSPC fraction exhibited decreased clonogenic survival in comparision to CMP, GMP and MEP subsets, in agreement with their increased apoptosis rate. Taken together, our results indicate that isolated human HSPCs are more susceptible to IR-induced cell death than CPs.

### IR-induced apoptosis in HSPCs is an ATM-dependent process that is counteracted by stroma

**Figure 2:**
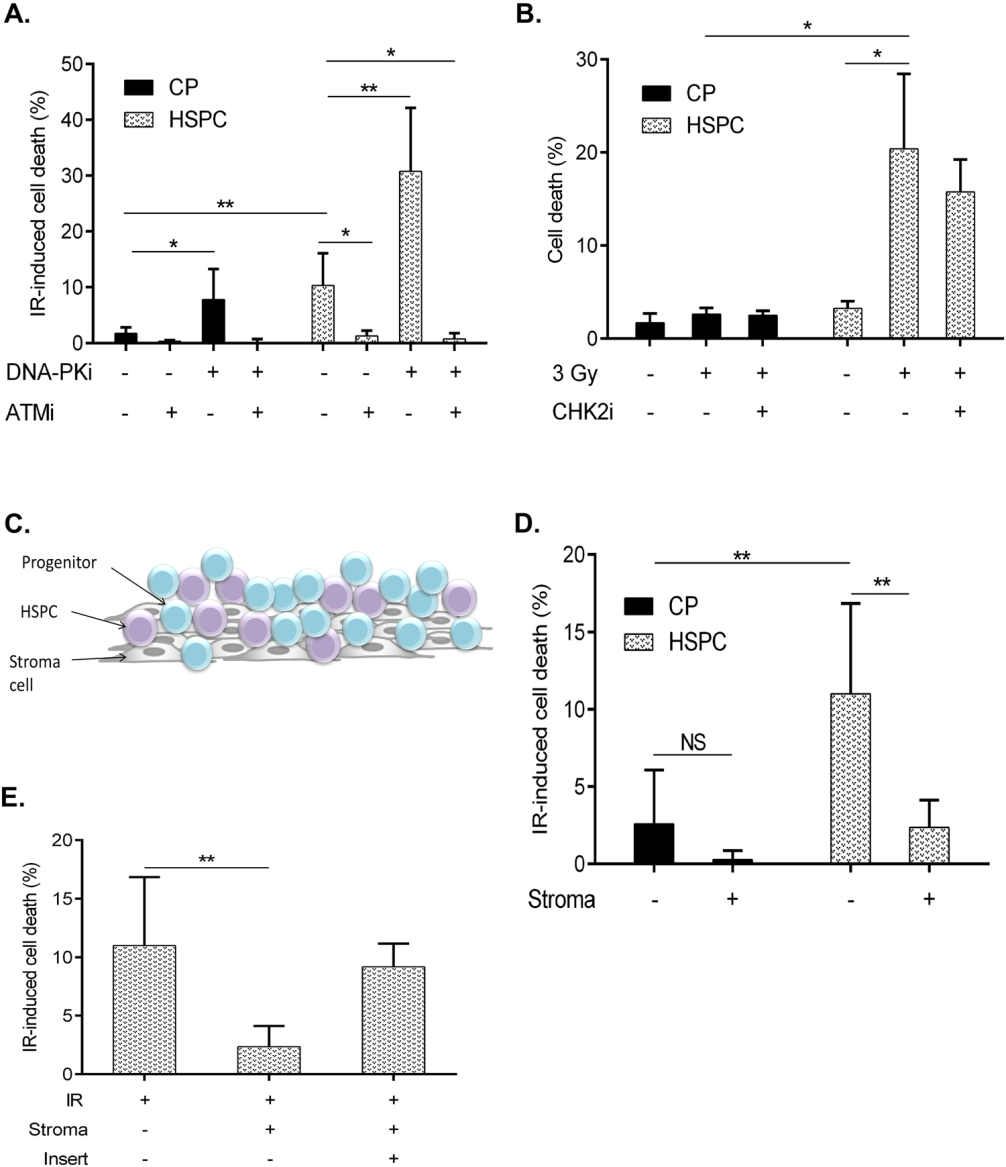
Regulation of IR-induced apoptosis in HSPCs and CPs. (**A)** Freshly isolated CD34^+^ cells were pre-treated with ATMi (Ku60019, 10 μM), DNA-PKi (Nu7441, 10 μM) for 1 h, irradiated with 3 Gy and analyzed for cPARP + cells in HSPC and CP fractions 6 h later.n ≥ 4 **(B)** Freshly isolated CD34^+^ cells were pre-treated with CHK2i (PV1019, 10 μM) for 1 h, irradiated with 3 Gy and analyzed for Annexin+ cells in HSPCs and CPs 6 h later. n = 3 **(C)** Schematic presentation of OP9M2 stroma CD34^+^ co-culture system. **(D)** Freshly isolated CD34^+^ cells were irradiated (3 Gy) or not and immediately plated on pre-established confluent layers of OP9M2 stromal cells for 6 hrs followed by Annexin^+^ cells analysis in HSPC and CP gates. IR-induced cell death was calculated to reduce the variability between CD34^+^ batches by subtracting the fraction of c-PARP^+^ or AnnexinV^+^ cells scored in the untreated sample from the same fraction in the IR sample n = 7 **(E)** CD34^+^ cells were plated on OP9M2 stroma as described in (D) in the presences or absence of pore (0.4 µm) membrane insert, n ≥ 3. Represented are mean values ± SD, *p < 0.05, **p < 0.01.

To identify factors that regulate the apoptosis of irradiated HSPCs, we utilized a pharmacological approach. Complementary RNA interference approach can not be applied in our experimental setting as we investigate freshly isolated human HSPCs and measure their immediate response (6–24 hrs post isolation) to DNA damage. Utilization of the genetic tools requires *ex vivo* culturing of HPSCs which inevitably leads to changes in their immunophenotype, cell cycle status, gene expression and most importantly, function. We inhibited key regulators of DSB-induced signaling using well characterized chemical inhibitors of ATM (Ku60019), DNA-PK (NU7741) and CHK2 (PV1019 and CHK2 inhibitorII). As can be seen in Supplementary Figure 2, ATM inhibition resulted in the reduced activating phosphorylation on ATM (P-Ser 1981) as well as on its direct target CHK2 (reduced P-Thr68-CHK2 signal)(Supplementary Figure 2A). DNA-PK inhibitor reduced Ser2056 autophosphorylation as a marker for DNA-PK activity and CHK2 inhibitors reduced CHK2 autophosphorylation activity on Ser 516 (Supplementary Figure 2B and C). Therefore, we confirmed that these kinase inhibitors indeed inhibit their intended targets. To reveal the role of ATM, DNA-PK and CHK2 kinases in the IR-induced cell death of HSPCs and CPs, CD34^+^ cells were pre-incubated with the described inhibitors before irradiation (Fig. 2). We found that blocking of ATM kinase significantly decreased the rate of apoptosis in HSPCs (Fig. 2A). In contrast, inhibition of DNA-PK activity dramatically increased HSPCs apoptosis after IR (Fig. 2A), underscoring the importance of DNA-PK for enhancing the resistance of these cells to genotoxic stress. Since we revealed that both ATM and DNA-PK kinases regulate IR-induced apoptosis in HSPCs, we investigated the epistatic relationships between them. Combined blocking of ATM and DNA-PK kinases prevented IR-induced apoptosis in HSPCs, similarly to the effect of ATM inhibition alone. Notably, DNA-PK inhibition also increased apoptosis rate in otherwise apoptosis-resistant CPs and combined inhibition of DNA-PK and ATM prevented apoptosis. These results suggest that ATM kinase is a positive regulator of DNA damage-induced apoptosis in HSPCs and CPs acting upstream of DNA-PK.

CHK2 kinase is one of the key ATM effectors implicated in DNA damage-induced apoptosis. To investigate its role in the IR-induced death of human HSPCs, we incubated CD34^+^ enriched cells with PV1019, a potent and highly specific CHK2 inhibitor^25^. Interestingly, we observed a similar proportion of apoptotic cells in HSPCs incubated with the solvent or CHK2 inhibitor, suggesting that CHK2 kinase activity is dispensable for IR-induced apoptosis in quiescent human HSPCs (Fig. 2B). No effect on the IR-induced apoptosis was observed using structurally different CHK2 kinase inhibitor (Chk2 inhibitor II, BML-277) (Supplementary Figure 3).

Bone marrow niche plays an important regulatory role in the maintenance of HSPCs and can influence their survival. To investigate the potential effect of bone marrow stromal cells on IR-induced HSPC apoptosis, we employed a co-culture system in which irradiated CD34^+^ cells were plated on top of the bone marrow stromal cells (OP9M2 line), capable of supporting the most primitive human HSPCs^19^ (Fig. 2C). Strikingly, co-culture with stroma strongly decreased IR-induced apoptosis in HSPCs compared with culture in growth medium alone (Fig. 2D). Importantly, the stroma-mediated protective effect was greatly attenuated when the physical contact between OP9M2 stroma cells and irradiated HSPCs was prevented by using a porous polycarbonate membrane (Fig. 2E). These results suggest that direct interaction between HSPCs and bone marrow stroma engages signaling pathways that can counteract the onset of IR-induced apoptosis.

Collectively, our analysis revealed that ATM and DNA-PK contribute to the rapid apoptosis onset of irradiated human HSPCs. Furthermore, contact with bone marrow stroma cells can counteract this process.

### HSPCs exhibit reduced NHEJ activity

**Figure 3:**
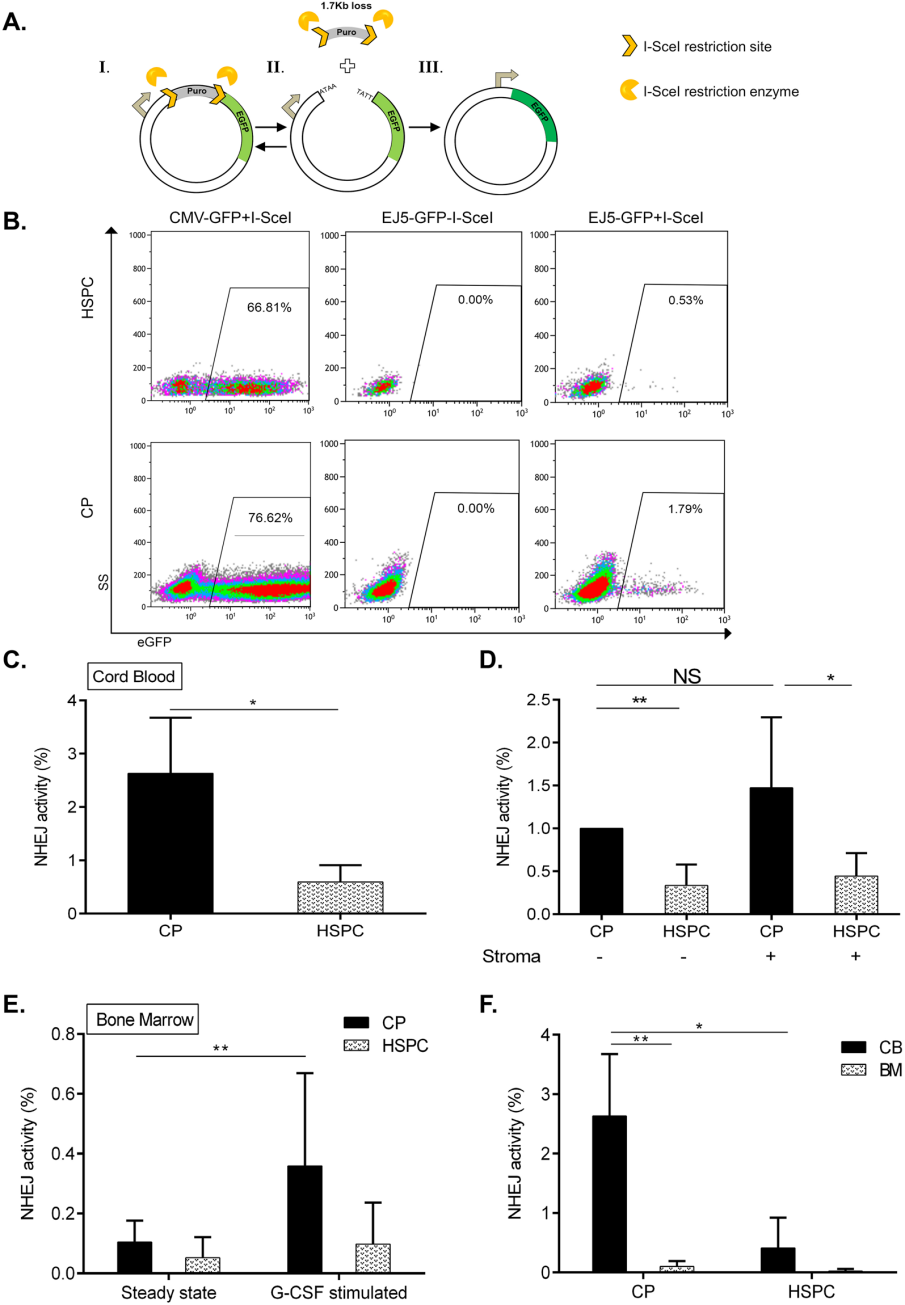
Analysis of DSB repair via NHEJ in CPs and HSPCs using a reporter system. **(A)** Schematic presentation of DSB repair substrate EJ5-GFP (I.) and I-*Sce*I endonuclease-mediated cleavage (II.), triggering NHEJ processes which restore the EGFP expression cassette (III.). **(B)** Representative flow charts of NHEJ activity and nucleofection efficiency in HSPCs and CPs. Freshly isolated CD34^+^ cells from cord blood were nucleofected with plasmid CMV-GFP (positive control for EGFP expression) or EJ5-GFP reporter with or without I-*Sce*I endonuclease expression plasmid and cultured for 24 h. Then, the proportion of viable EGFP + cells in HSPC- and CP- fractions gated as described in Fig. 1A was analyzed by flow cytometry. **(C)** NHEJ capacity of CP- and HSPC- enriched fractions isolated from cord blood (n = 3). **(D)** Nucleofected CD34^+^ cells from cord blood were plated in wells containing medium only or stroma layers for 24 hrs, followed by NHEJ capacity assessment in CP- and HSPC- enriched fractions. **(E)** Human bone marrow samples obtained from G-CSF treated (10 ug/kg for 5 days prior to harvesting) (n = 2) or steady-state control donors (n = 4) were nucleofected and processed for NHEJ activity assessment as described in Fig. 1B. NHEJ frequencies were individually normalized to the nucleofection efficiencies in each subpopulation and experiment. **(F)** Comparison of NHEJ frequencies of HSPCs and CPs derived from cord blood versus untreated bone marrow based on measurements presented in **(C)** and **(E)**. Represented are mean values ± SD; *p value < 0.05, **p value < 0.01, NS, non-significant.

The efficiency and fidelity of DSB repair in HSPCs (after IR) are critical for the survival and prevention of potentially leukemogenic aberrations. NHEJ is the major DSB repair pathway that is active in quiescent HSPCs. To investigate the capacity of NHEJ-mediated DSB repair in HSPCs, we used the reporter assay system EJ5-EGFP^26^. In this reporter, a 1.7 kb spacer, which is flanked by two I-*Sce*I restriction endonuclease sites, separates *EGFP* cDNA from the promoter and thus prevents EGFP expression. I-*Sce*I expression introduces two tandem DSBs in the plasmid that can be repaired via multiple sub-types of NHEJ, resulting in EGFP expression that can be measured as the percentage of EGFP^**+**^ cells (^26^ and Fig. 3A). Using this assay, we found that the HSPC-enriched fraction exhibited a 2–6-fold lower total NHEJ activity as compared with CP (Fig. 3B,C). Notably, HSPCs and CPs exhibited similar nucleofection efficiencies and no detectable EGFP^**+**^ cell background in the absence of I-*Sce*I (Fig. 3B).

To determine whether bone marrow stroma can alter the NHEJ capacity of HSPCs and CPs, we utilized our co-culture system. As shown in Fig. 3D, the HSPC-enriched fraction exhibited 2–5-fold lower NHEJ compared with CPs also with stroma support. As some DNA repair factors are under cell cycle control we analyzed the cell cycle status using combined Ki-67/Hoechst 33342 staining of HSPCs and CP subtypes isolated by flow sorting from cord blood (Supplementary Figure 4). Although HSPCs contained a higher proporion of Ki67^−^ cells relatively to CPs, none of the cells were in S/G2M stages of cell cycle at the time of DNA repair analysis. These results suggest that the difference in NHEJ activity between HSPCs and CPs is not due to their cell cycling changes.

To further substantiate these findings, we investigated NHEJ activities in HSPCs and CPs derived from human bone marrow (BM), i.e., in adult HSPCs (Fig. 3E). In the steady-state BM we detected very low NHEJ activity in HSPC and CP subpopulations compared with their respective neonatal counterparts. To determine whether negligibly low NHEJ activities of BM-derived HSPCs and CPs can be affected by the proliferation status of the cells, we used BM samples obtained from Granulocyte-Colony Stimulating Factor (G-CSF)-treated healthy donors. Strikingly, G-CSF stimulation led to a 3-fold higher NHEJ activity in the CP fraction, whereas no change was observed in HSPCs (Fig. 3E). These results revealed that human neonatal HSPCs have a low NHEJ capacity in comparison with their more mature progeny irrespective of the stroma interaction. G-CSF stimulation of human adult BM up-regulates NHEJ selectively in the CP fraction. Taken together, these results indicate a relative NHEJ repression in both neonatal and adult HSPCs, as compared with their respective CPs (Fig. 3F).

### Alternative-EJ repair is limited in HSPCs

**Figure 4:**
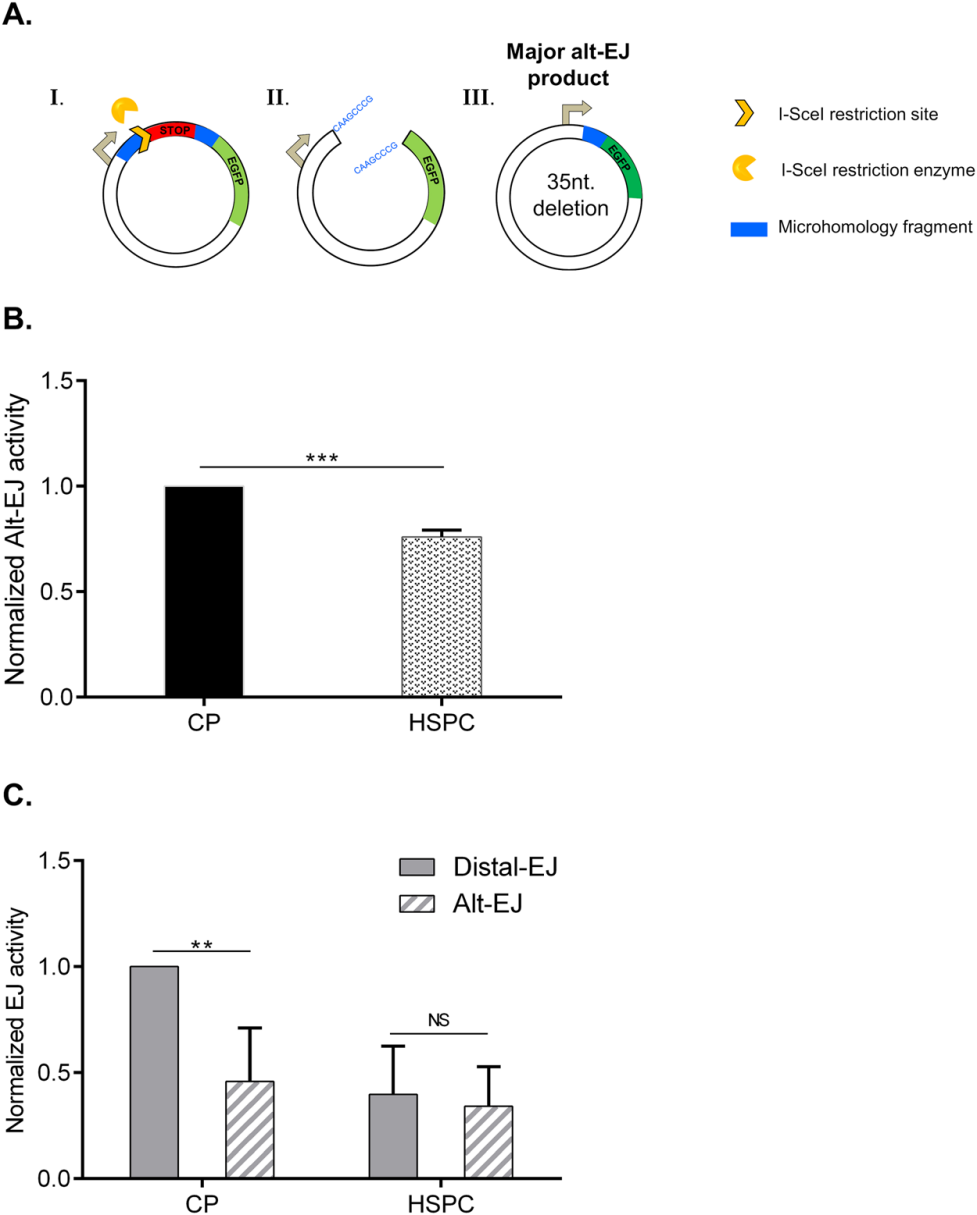
Alt-EJ activity and its regulation in CPs and HSPCs. **(A)** Schematic presentation of DSB repair reporter EJ2-GFP (I.), repair intermediate induced by I-*Sce*I endonuclease (II.) and EGFP + product obtained via Alt-EJ (III.)^26^. **(B)** Freshly isolated CD34^+^ cells were nucleofected with EJ2-GFP reporter together with I-*Sce*I expression plasmid and cultured for 24 h. Then, the fraction of EGFP + cells in viable CPs and HSPCs cell fractions gated as described in Fig. 1A was analyzed by flow cytometry, n = 3. **(C)** Comparison of NHEJ and Alt-EJ capacity in CPs and HSPCs based on measurements presented in Figs. 3C and 4B. The proportion of Alt-EJ events (measured by EJ2-GFP assay) were calculated by dividing EJ2-GFP readout on the EJ5-GFP readout for the same cell fraction. Represented are mean values ± SD, **p value < 0.01, ***p < 0.001, NS; non-significant.

NHEJ is a major pathway of DSB repair. In that pathway the broken DNA ends can be ligated without the need of extensive homology. Indeed, EJ5-GFP based detection of NHEJ capacity can involve also some mutagenic NHEJ activity component as it comprises detection of both classical-NHEJ and Alt-NHEJ^26^. Mutagenic outcome involves the loss of the microhomology sequences flanking the DSB which are a hallmark of genetically distinct Alt-NHEJ^6,7^. To investigate the activity of Alt-NHEJ in human HSPCs, we used the EJ2-EGFP repair reporter construct, in which the EGFP^+^ repair outcome upon I-*Sce*I expression requires micro-deletion of the stop codon spacer (35nt) and the annealing of 8nt-long homologous sequences that restore the *EGFP* coding frame^26^ (Fig. 4A). I-*Sce*I-mediated DSB formation in the EJ2-EGFP reporter resulted in EGFP^+^ products in both CP and HSPC cells. Interestingly, we observed 30% lower erroneous repair activity in HSPCs compared with CPs (Fig. 4B). Next, we compared the relative utilization of total NHEJ and Alt-EJ pathways in CPs and HSPCs. As shown in Fig. 4C, CPs had a twofold higher total NHEJ activity relative to Alt-EJ, whereas HSPCs had a similar capacity to generate repair products using total- and Alt-EJ reporters. Collectively, these results indicate that HSPCs might utilize a distinct balance of error-free and error-prone DSB repair mechanisms than their committed progeny.

### Attenuated DNA damage response in human quiescent HSPCs

**Figure 5:**
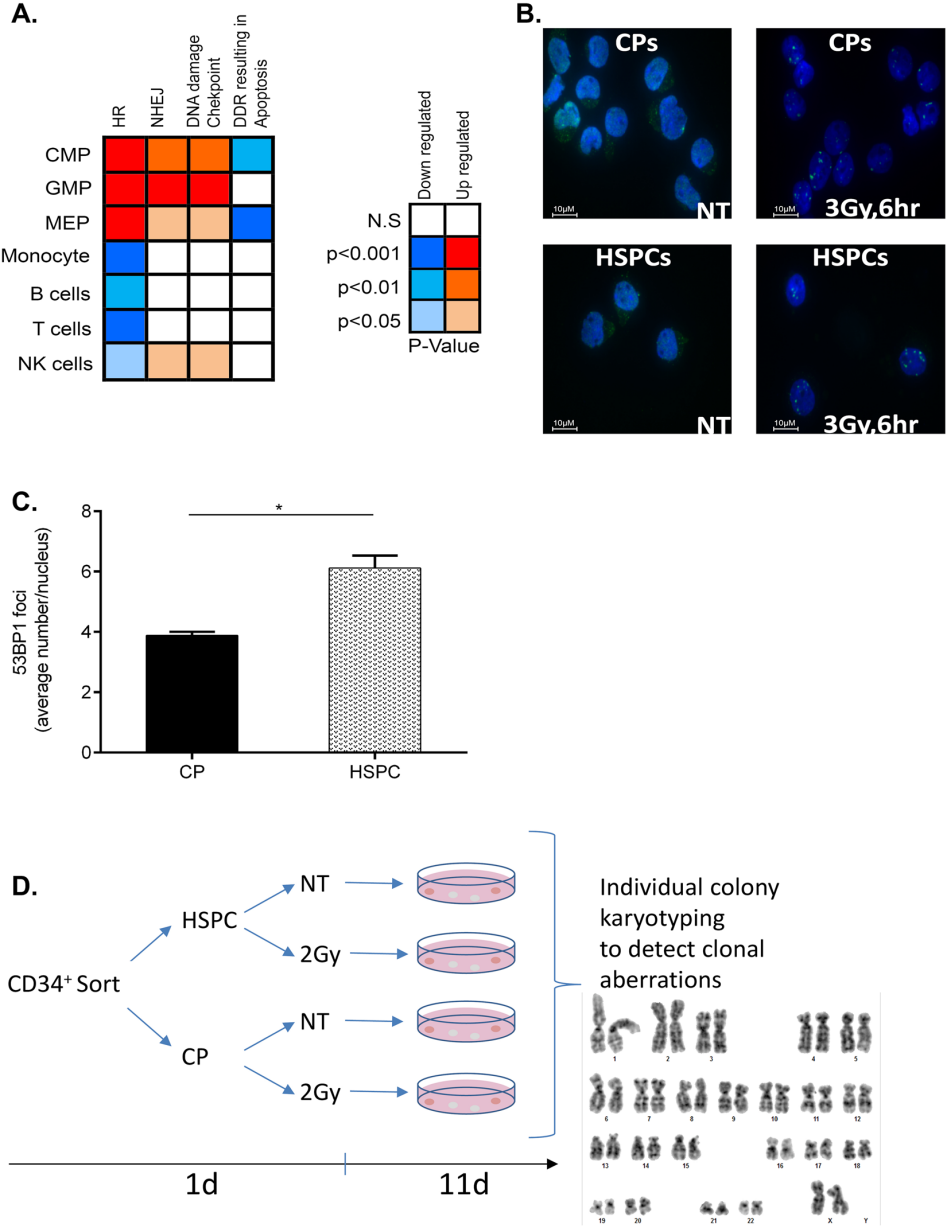
DNA repair and DDR gene expression, DSB marker and genome stability analyses. **(A)** left panel: relative expression of transcripts related to DNA repair, DDR checkpoint and apoptosis pathways in CPs (CMP, GMP and MEP) and mature blood-derived cells (B, T and NK) in comparison to HSPCs colored accordingly to the significance of the changes. right panel: color scheme description with blue colors for decreased expression and red colors indicative of increased expression relative to HSPCs along with the corresponding p values. **(B)** CD34^+^ cells were sorted into HSPC and CP fractions. Representative immunofluorescence staining visualized for 53BP1 (green) and DNA (blue) in HSPCs and CPs 6 hr after 3 Gy IR. **(C)** Average number of 53BP1 foci per cell in CPs and HSPCs. n = 3 experiments; Represented are mean values ± SD; *p < 0.05. At least 100 nuclei were analyzed in each experiment. **(D)** Workflow for the karyotype analysis of irradiated HSPCs and CPs. Freshly isolated CD34^+^ cells were sorted for HSPC and CP fractions, exposed or not to IR (2 Gy) and then plated at low density in methylcellulose to allow colony formation. Individual colonies were isolated for metaphase preparation and karyotype analysis (Table 1, and Supplementary Table 1).

Our observations of decreased NHEJ activities as well as enhanced susceptibility of HSPCs to IR-induced apoptosis in comparison with CPs raised the possibility that DNA repair regulators and/or cell death genes might be differentially expressed in these subpopulations. To examine this idea at the genome-wide scale, we performed a bioinformatics analysis of the transcript expression of genes involved in NHEJ, HR, DNA damage checkpoint, and apoptosis. First, we extracted and normalized the expression values^22^ of 77 genes annotated to the above pathways extracted from studies that performed global transcriptional profiling of human HSPCs and downstream progenitors^27–32^. Next, we calculated the average expression value for each pathway as an indicator of its activity in the various subpopulations, and determined the statistically significant differences between them. This analysis revealed an upregulation of HR, NHEJ, and DNA damage checkpoint pathways as a whole, in all three types of CPs (CMPs, MEPs, and GMPs) compared with HSPCs (Fig. 5A). In contrast to these gene sets, the apoptosis pathway defined by the gene set: “DDR resulting in apoptosis” displayed significantly reduced expression levels in CMPs and MEPs relative to HSPCs. Only the HR DNA repair pathway was further downregulated in all mature cell types relative to HSPCs. This analysis of global DDR pathway activity is in agreement with our experimentally observed decrease in NHEJ capacity and the increased apoptosis sensitivity of human HSPCs.

Decreased expression of NHEJ and DDR gene transcripts can lead to appreciable changes in DSB-associated signaling and repair, as observed here. To validate this hypothesis *in situ*, we quantified IR-induced 53BP1 foci. 53BP1 protein promotes NHEJ, rapidly recruited to the sites of DSBs and it is widely used as a marker of DSB repair progress. Six hours after IR, HSPCs displayed significantly higher numbers of 53BP1 foci compared with CPs (6.1 versus 3.8 foci/nucleus, respectively), as well as 12 h post IR (Supplementary Figure 5) suggesting delayed DSB repair or persistent DNA damage signaling (Fig. 5B,C), in agreement with sustained gH2AX foci in HSPCs as reported elsewhere^17^. Of note, we observed no difference in gH2AX foci induction between HSPCs and CPs at 1 hr post IR^17^. Taken together, these results strengthen our findings from reporter assays indicating lower NHEJ capacity, as well as, higher sensitivity to IR-induced cell death.

### Induction of clonal chromosomal aberrations in irradiated HSPCs

Chromosomal aberrations that result from misrepaired DSBs are the hallmarks of radiation exposure and genomic instability that together can lead to hematological malignancies or bone marrow failure. High sensitivity to IR-induced apoptosis and decreased DSB repair capacity of HSPCs can affect their susceptibility to IR-induced chromosomal aberrations. To test this hypothesis, we assessed the frequency of spontaneous and IR-induced chromosomal abnormalities in HSPCs and CPs at the clonal level using cytogenetic analysis. We assumed that misrepaired DSB in the surviving individual HSPCs or CPs will be inherited by their respective progeny, which can be directly isolated for karyotyping from individual colonies generated in methylcellulose^21^ (Fig. 5D, Table 1). Using G-banding technique, we analyzed multiple metaphases from each colony generated by sorted HSPCs or CPs. We found clonal aberrations such as translocations in 10% of the colonies formed by HSPCs (2 out of 20 colonies investigated), whereas 8.1% of the colonies generated by irradiated CPs contained clonal aberrations (3 out of 37). No chromosomal abnormalities were detected in the progeny formed by unirradiated HSPCs and CPs (Table 1, Supplementary Figure 5, and Supplementary Table 1). Thus, significant fraction of human HSPCs that survived sublethal irraidation exhibits gross chromosomal aberrations. Collectively, our multifacet analysis of human HSPCs’ response to DNA damage indicate that these primitive cells are not distinctively protected from the onset of genome instability.

**Table 1:** Clonal chromosomal aberrations in the progeny of individual HSPC and CP.

	CP		HSPC	
IR	—	2 Gy	—	2 Gy
# Colonies	6	37	6	20
# Clonal aberrations	0	3	0	2
% Aberrant colonies	0	8.1	0	10

Full karyotype analysis for each colony can be found in Supplementary table 1.

Table 1 presents summary of three independent sorting and karyotyping experiments using cord blood preparations from separate donors.

^1^Clonal chromosomal aberration frequency (%) was calculated by dividing the number of

colonies that carried stable aberration by the total number of colonies analyzed from the same group

^2^Representative metaphases for each group can be found in Supplementary Figure 5.

## Discussion

Accumulation of genomic aberrations in human HSPCs can severely impair their function or promote their transformation to leukemia-initiating cells. Here we provided evidence for both the cytotoxic and mutagenic consequences of DSB formation in quiescent HSPCs and analyzed the role of bone marrow niche in causing these effects.

Although bone marrow cells are considered to be the most radiosensitive cells in the body^33^, little knowledge exists on IR-induced cytotoxic and mutagenic effects specifically in human HSPCs. In this work we revealed that irradiated HSPCs underwent ATM-dependent but CHK2-independent apoptosis, which is surprising finding, given that IR-induced apoptosis in murine and human lymphocytes requires CHK2^34–36^. On the other hand, unrepaired DSBs generated during VDJ recombination in G0/G1 lymphocytes trigger CHK2-independent apoptosis^37^. Low expression of CHK2 or its sequestration to centrosomes, which recently has been shown for murine HSPCs^23^, can plausibly explain CHK2-independent apoptosis of human HPSCs.

In the body, HSPCs reside in a special stromal microenvironment, which is thought to regulate their differentiation, self-renewal, migration, and survival^38^. Potent attenuation of IR-induced apoptosis by co-culture on stroma cells, which was revealed here, emphasizes that cues from the microenvironment, rather than an intrinsic propensity for apoptosis, can affect the immediate survival of HSPCs. Indeed, this notion can reconcile seemingly contradictory results regarding the high^23,39–41^ and low sensitivity of murine HSPCs to IR-induced apoptosis^15,16^. Notably, pre-incubation of HSPCs with stroma was not required to obtain a completely protective effect. This may argue in support of the rapid post-translational changes occurring inside HSPCs, which block apoptosis onset. Indeed, stabilization of pro-survival (e.g., Mcl1, Bcl-xL) or enhanced degradation of pro-apoptotic (e.g., Bim) proteins by extrinsic factors was reported elsewhere^42–45^. Although the exact mechanism by which bone marrow stroma blocks IR-induced apoptosis in HSPCs is currently unknown, OP9M2 stroma serves as a niche-mimicking platform for further detailed evaluation of DDR mechanisms in human hematopoietic tissues.

Knowledge of the activity and regulation of NHEJ pathways, which are ultimately responsible for DSB repair in quiescent HSPCs, can help in determining the mutagenic outcome of the genotoxic insult^15,24,46,47^. In this study use of DNA repair reporters revealed that human HSPCs have a 2–6-fold lower frequency of NHEJ events relative to the fraction of CPs. This DNA repair activity, in the context of multiple and simultaneously arising DSBs, such as those introduced by IR and Topoisomerase inhibitors, can lead to chromosomal translocations^6^. In line with our findings obtained with live HSPCs, Zhou and coworkers demonstrated a decreased NHEJ capacity in the nuclear extracts from CD34^+^38^−^ relative to CD34^+^38^+^ using linearized plasmids as the substrates^18^. Notably, since most of the experiments in the current study were performed with neonatal cells, whereas the group of Zhou employed adult BM hematopoietic cells^18^, we can conclude that the decreased NHEJ capacity of human HSPCs is an age-independent phenomenon. Moreover, low NHEJ capacity cannot merely be associated with the quiescent status of HSPCs, because it also remained low when we compared cycling HSPCs and CPs from G-CSF-stimulated BM. Consistently, in studies that analyzed quiescent HSPCs versus cycling CPs^18^ and *ex vivo*-cultivated CD34^+^ cells with lymphocytes^48^ compromised NHEJ activities were detected in HSPCs and CD34^+^ cells, respectively. Altogether, decreased NHEJ activity was found in the more primitive hematopoietic fraction. Importantly, we revealed that G-CSF stimulation of human BM led to NHEJ upregulation exclusively in the CP compartment, providing a plausible explanation for the genomic instability reported for lymphocytes but not for HSPCs in donors receiving G-CSF as part of the clinical HSPC mobilization^49–51^.

In contrast with the 2–6-fold difference in total NHEJ capacity, Alt-EJ activity was more moderately reduced in HSPCs compared with CPs, namely, by 30%. Similarly, in human CD34^+^ cells, NHEJ was one order of magnitude lower but Alt-EJ activities were similar when compared with peripheral blood lymphocytes^48^, thus supporting the notion that canonical NHEJ capacity rises with the degree of differentiation in hematopoietic cells. In agreement with this notion and our own results, we observed a broad transcriptional attenuation of DSB repair and checkpoint pathways as well as a distinct amplification of the DDR-related apoptosis gene set in HSPCs relative to their progeny. Reduced expression of multiple DNA repair genes in CD34^+^38^−^ cells^52^ and decreased expression of Ligase 4 and ATM, in particular^18^, could explain the diminished NHEJ capacity and persistent 53BP1 foci. Decreased ATM expression can also impinge on the reduced CHK2 activation, which is consistent with its uncoupling from apoptosis, as we reported here. Although the mechanisms underlying the differential expression pattern of DDR sets remain largely unknown, the interconnected signaling networks regulating HSPC quiescence, on the one hand, and DNA repair gene expression, on the other (e.g., NF-Kb, PI3K/Akt, and others), should be considered^24,48,53^.

Signaling cascades differentially wired by DSBs in HSPCs vs. more differentiated cells can greatly influence cellular survival as well as the mutagenic outcome of the repair^48,54^. Our genome stability analysis of irradiated HSPCs vs. CPs revealed that surviving HSPCs were not specifically protected from acquiring chromosomal aberrations. Furthermore, these aberrations were transmitted to the daughter cells of the colony, thus potentially increasing the chances of pre-leukemia HSPCs to evolve. On the other hand, the long-term self-renewal potential of these aberrant HSPCs remains to be investigated. Moreover, it is acknowledged that in addition to apoptosis, IR also can induce precipitous differentiation and senescence pathways that can strongly limit irradiated HSPCs’ long-term regenerative potential^55–57^. As regulators and markers of these additional DDR pathways emerge, it will be necessary to determine their involvement in controlling human HSPCs genome integrity and stability.

In conclusion, our data revealed two mechanisms that can contribute to the cytotoxic and mutagenic effects in human HSPCs upon exposure to exogenous DNA damage. Although bone marrow niche can mitigate the cytotoxic effect of DNA damage, the inherent deficiency in the DSB DNA repair pathways of the surviving HSPCs might provide a potential mechanism by which radiation-induced leukemogenesis occurs.

## Electronic supplementary material

Supplementary information
