# Supplementary material for: Attenuated DNA damage responses and increased apoptosis characterize human hematopoietic stem cells exposed to irradiation
Source: Sci Rep. 2018 Apr 17;8:6071. doi: 10.1038/s41598-018-24440-w (PMC5904119; doi:10.1038/s41598-018-24440-w)
Supplement: Supplementary file 1 — Supplementary information [file 41598_2018_24440_MOESM1_ESM.pdf]

Supplementary Information

Attenuated DNA damage responses and increased apoptosis characterize human hematopoietic stem cells exposed to irradiation

Running head: DDR of human Hematopoietic Stem Cells

Shahar Biechonski[1,2], Leonid Olender[1,2], Adi Zipin-Roitman[1,2], Muhammad Yassin[1,2], Nasma Aqaqe[1,2], Victoria Marcu-Malina[3], Melanie Rall-Scharpf [4], Magan Trottier[5,6], M. Stephen Meyn[5,7], Lisa Wiesmüller[4], Katia Beider[8], Yael Raz[1,2,9], Dan Grisaru[2,9], Arnon Nagler[8], and Michael Milyavsky[1, 2,#]

[1]Department of Pathology, [2]Sackler Faculty of Medicine, Tel-Aviv University, Tel-Aviv, 69978, Israel

[3]Cytogenetic Unit, Laboratory of Hematology, Chaim Sheba Medical Center, Tel-Hashomer, Israel.

[4] Department of Obstetrics and Gynecology, Gynecological Oncology, University of Ulm, Prittwitzstrasse 43, Ulm, Baden-Wuerttemberg 89075, Germany.

[5] Department of Molecular Genetics, University of Toronto, ON M5G 1L7, Canada

[6] School of Medicine and Public Health, University of Wisconsin, Wisconsin, USA

[7] Hematology Division, Chaim Sheba Medical Center, Tel-Hashomer, Israel.

[8] Department of Obstetrics and Gynecology, Gynecologic Oncology Division, Lis Maternity Hospital, Tel Aviv Sourasky Medical Center, Tel-Aviv 64239, Israel.

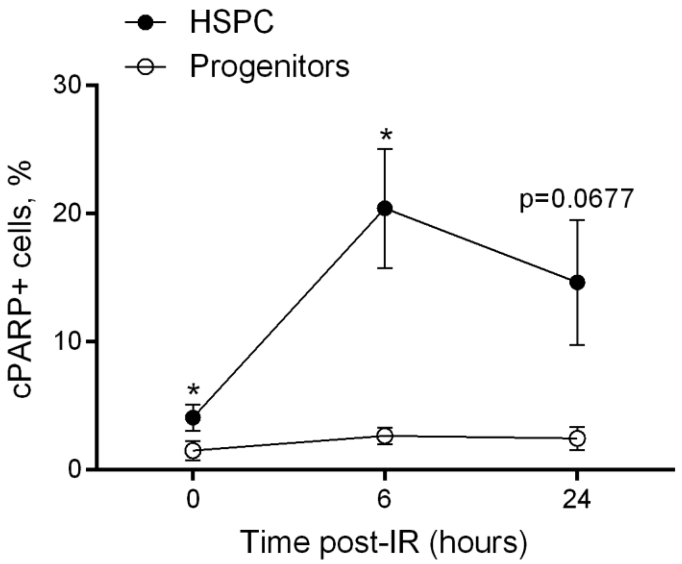

Supplementary Figure 1. Kinetics of cPARP induction after IR. CD34+ enriched cells were irradiated (3Gy), harvested and analyzed for cPARP induction by flow cytometer in CP and HSPC subpopulations at the indicated time points. No significant changes in the surface marker expression was evident during this time course (Supplementary Figure 7).

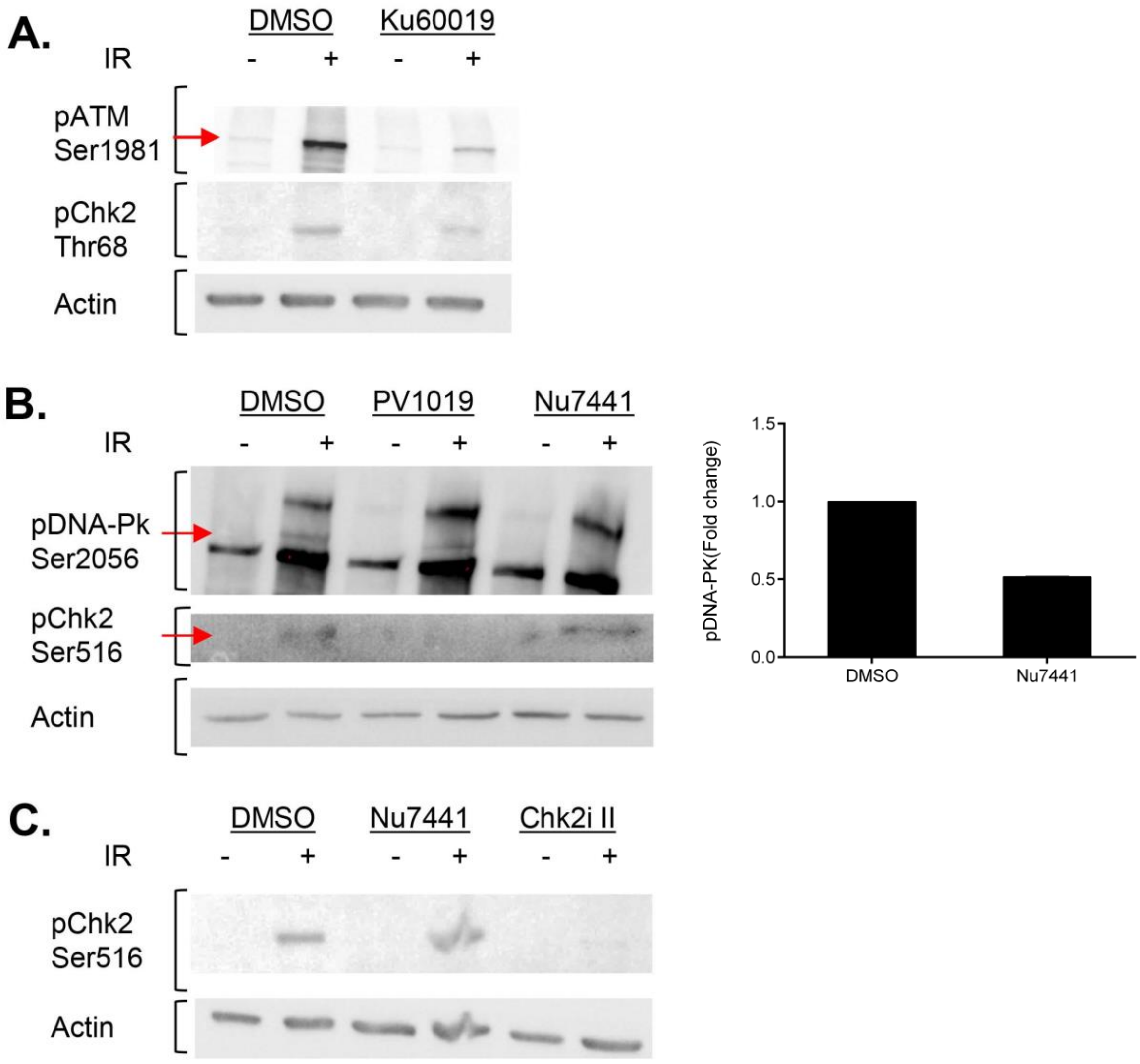

Supplementary Figure 2. DDR inhibitors validation. OCI-AML2 human leukemia cell line was pre-incubated with the indicated inhibitors (10uM) for 1hr followed by IR (10Gy). One hour after IR cells were harvested for Western blotting analysis of activated ATM and its target CHK2 (A), activated DNA-PK and activated CHK2 (B and C). Quantification of pDNA-Pk (Ser2056) protein levels. Protein band intensity (labeled

by red arrow) was quantified for each treatment and normalized to the corresponding loading controls. Actin protein expression was used as a loading control. n=2.

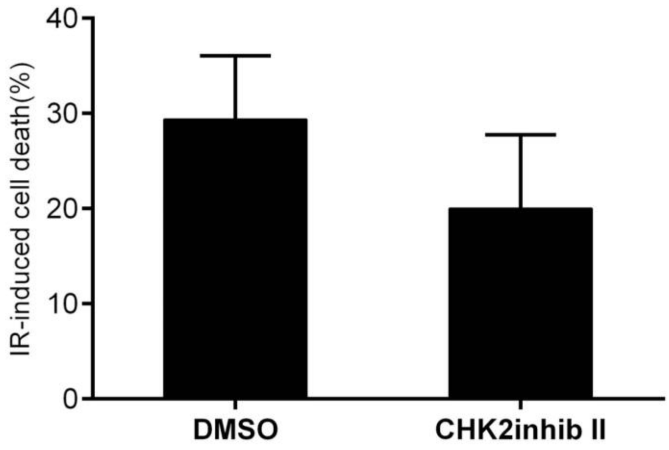

Supplementary Figure 3. CHK2 activation is dispensable for IR-induced apoptosis in HSPCs. Freshly isolated CD34+ cells were pre-treated with CHK2 inhibitor II (BML-277, 10uM) for 1h, irradiated with 3Gy and analyzed for Annexin+ cells in HSPCs. n=3.

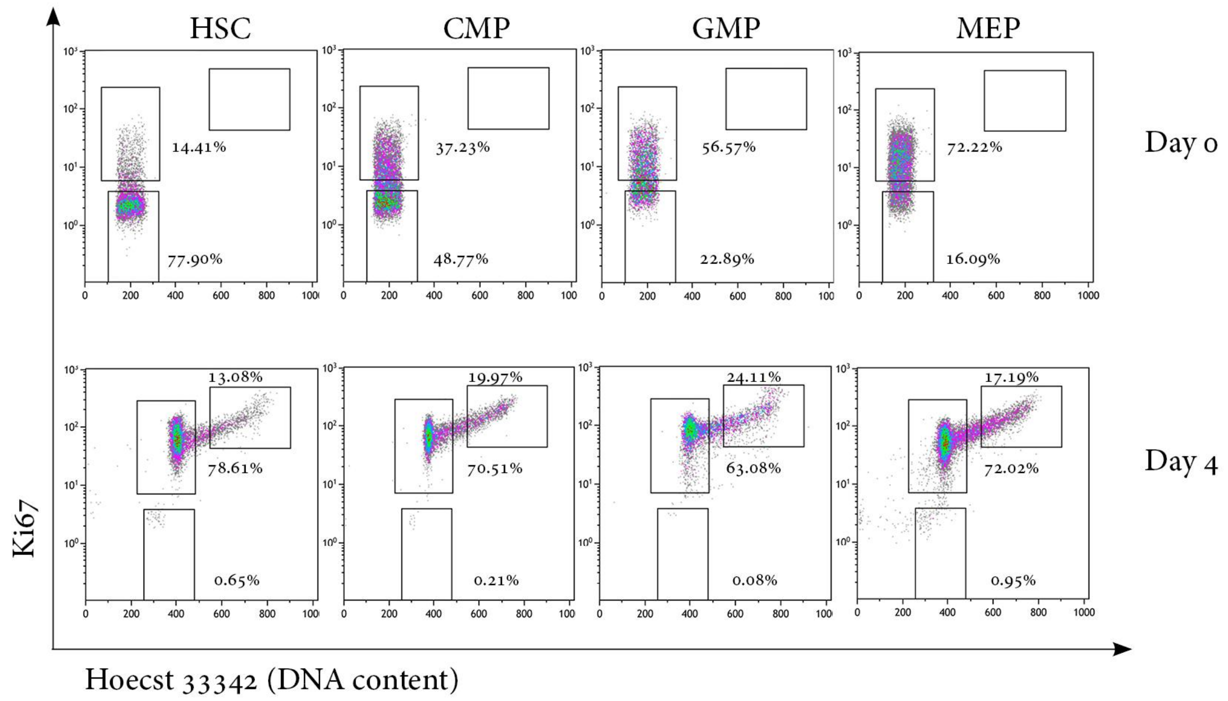

Supplementary Figure 4. Cell cycle analysis of HSPCs and CPs. Freshly isolated CD34^{+} cells were sorted to obtain HSPCs (CD34^{+}38$^{-/low}$CD45RA^{-}CD90^{+}), CMP (CD34^{+}38^{+}CD45RA^{-}CD135^{+}), GMP (CD34^{+}38^{+}CD45RA^{+}CD135^{+}) and MEP(CD34^{+}38^{+}CD45RA^{-}CD135^{-}) fractions and were either fixed immediately or plated in medium supplemented with cytokines for 4 days followed by fixation. Then, cells from both time points were stained with anti-Ki-67 antibody conjugated with FITC and their DNA quantity was assessed by Hoechst 33342. Percentage of cells in G0 (Ki-67^{-}) and G1 (Ki-67^{+}) stages of cell cycle in different cell fractions is indicated. CD34+ cells were cultured for 4 days under the same conditions as described for nucleofection.

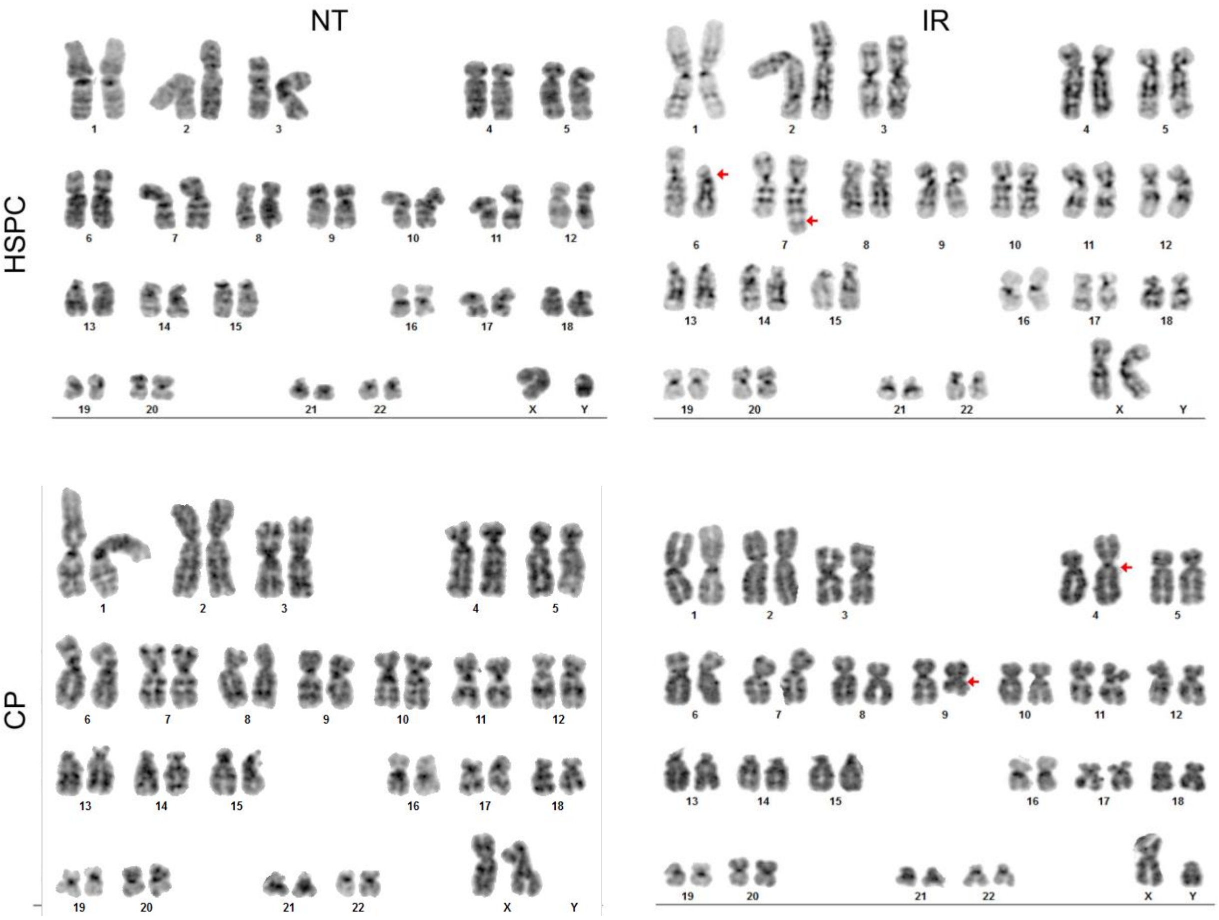

Supplementary Figure 5. Representative karyotypes of HSPCs and CPs.

Metaphases were prepared from individual colonies and processed for karyotyping using Giemsa banding technique. Representative karyotypes of un-irradiated HSPC (46,XY), un-irradiated CP (46,XX), irradiated HSPC (46,XX t(6;7) clonal aberration). Irradiated CP (46,XY t(4;9), clonal). Red arrows point to the reciprocal translocation breakpoints. At least 3 metaphases of a colony carried identical stable aberration as shown in the translocation examples to be considered clonal event.

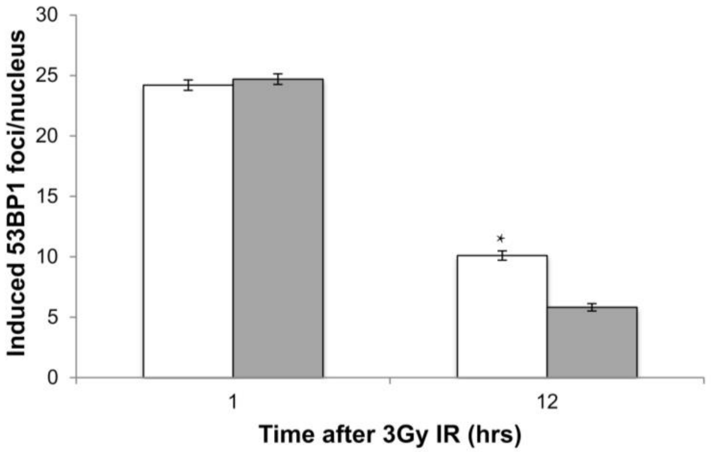

Supplementary Figure 6. Mean number of IR-induced 53BP1 foci in HSPCs and CPs. 53BP1 foci 1 and 12 hours after exposure to IR. Induced foci were calculated by subtracting mean foci in un-irradiated control cells from irradiated cells for each replicate. Foci numbers are shown as averages of 100-184 cells scored from 3-4 replicates with SE indicated. Asterisks indicate $p<0.0001$ by student's *t*-tests done pair-wise between populations at 1 hour and pair-wise between populations at 12 hours post-IR.

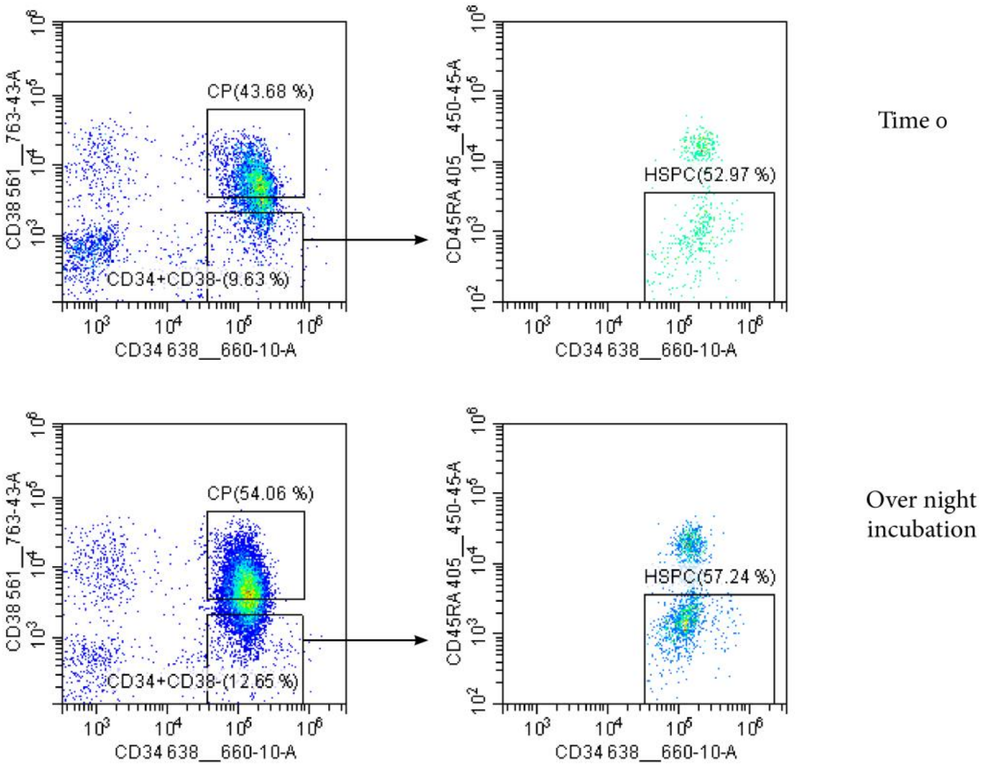

Supplementary Figure 7. Stable CD34, CD38, CD45RA expression during one day ex vivo culture. CD34[+] cells were enriched from CB followed by CD34, CD38 and CD45RA staining (time 0)). Cells were cultured in SFEM media with cytokines for 16 hrs and then analyzed for CD34,CD38 and CD45RA expression (lower plot).

Supplementary Table 1.

Karyotype analysis of individual colonies formed by sorted HSPC and CP exposed or not to ionizing radiation (2Gy)

Colony origin and treatment	Colony identifier	Metaphases on slide (n)	Metaphases analysed (n)	Karyotype
HSPC_NT	1B	12	3	(46,XX)
HSPC_NT	2B	11	2	(46,XX)
HSPC_NT	3B	32	4	(46,XX)
HSPC_NT	4B	59	4	(46,XX)
HSPC_NT	2C	39	3	(46,XX)
HSPC_NT	3C	>50	1	(46,XY)
HSPC_2Gy	6B	72	5	(46,XX)
HSPC_2Gy	7B	35	4	(46,XX)
HSPC_2Gy	8B	52	4	(46,XX)
HSPC_2Gy	9B	44	3	(46,XX)
HSPC_2Gy	10B	66	3	(46,XX)t(6;7)clonal
HSPC_2Gy	11B	38	3	(46,XX)
HSPC_2Gy	12B	33	3	(46,XX)
HSPC_2Gy	13B	31	3	(46,XX)
HSPC_2Gy	14B	28	4	(46,XX)t(10;17)clonal

HSPC_2Gy	15B	46	3	(46,XX)
HSPC_2Gy	5C	48	1	(46,XX)
HSPC_2Gy	7C	33	2	(46,XX)
HSPC_2Gy	9C	>50	4	(46,XX)
HSPC_2Gy	21E	3	1	(46,XX)
HSPC_2Gy	22E	4	1	(46,XX)
HSPC_2Gy	24E	5	1	(46,XX)
HSPC_2Gy	28E	50	4	(46,XX)
HSPC_2Gy	29E	>200	3	(46,XY)
HSPC_2Gy	30E	>200	4	(46,XX)
HSPC_2Gy	31E	>200	4	(46,XY)
HSPC_2Gy	32E	>200	4	(46,XY)
CP_NT	16B	29	3	(46,XX)
CP_NT	18B	28	3	(46,XX)
CP_NT	1D	>50	3	(46,XX)
CP_NT	2D	>50	3	(46,XY)
CP_NT	3D	>50	3	(46,XX)
CP_NT	13C	45	2	(46,XX)
CP_2Gy	19B	30	3	(46,XX)
CP_2Gy	20B	32	4	(46,XX)

CP_2Gy	21B	30	3	(46,XX)
CP_2Gy	22B	10	3	(46,XX)
CP_2Gy	23B	44	3	(46,XX)
CP_2Gy	24B	26	2	(46,XX)
CP_2Gy	25B	8	2	(46,XX)
CP_2Gy	27B	2	2	(46,XX)
CP_2Gy	28B	52	2	(46,XX)
CP_2Gy	6D	>50	3	(46,XY)
CP_2Gy	7D	>50	3	(46,XX)t(14;14)clonal
CP_2Gy	8D	>50	3	(46,XX)
CP_2Gy	9D	>50	3	(46,XX)
CP_2Gy	10D	>50	4	(46,XX)
CP_2Gy	11D	>50	3	(46,XY)
CP_2Gy	12D	>50	7	(46,XY)t(4;9)clonal
CP_2Gy	13D	>50	3	(46,XX)
CP_2Gy	14D	>50	1	(46,XX)
CP_2Gy	15D	>50	3	(46,XX)
CP_2Gy	17D	>50	1	(46,XX)
CP_2Gy	18D	>50	3	(46,XX)
CP_2Gy	19D	>50	3	(46,XX)
CP_2Gy	20D	>50	2	(46,XY)

CP_2Gy	59E	51	4	(46,XY)
CP_2Gy	61E	14	1	(46,XY)
CP_2Gy	62E	35	4	(46,XY)
CP_2Gy	63E	3	1	(46,XY)
CP_2Gy	64E	42	4	(46,XY)
CP_2Gy	65E	40	3	(46,XY)
CP_2Gy	66E	44	4	(46,XY)t(2;22)clonal
CP_2Gy	67E	26	2	(46,XX)
CP_2Gy	68E	21	4	(46,XY)
CP_2Gy	69E	20	3	(46,XX)
CP_2Gy	70E	36	5	(46,XX)
CP_2Gy	71E	43	3	(46,XX)
CP_2Gy	72E	13	3	(46,XY)

HSPC, Hematopoietic Stem and Progenitor Cell

CP, Committed Progenitor Cell

Supplementary Table 2. List of antibodies used

Antibody	Working dilution	Vendor, catalog number, clone	Notes
**Surface Antigens labeling**			
CD34	1:100	Beckman Coulter,IM2472, 581	
CD38	1:100	Beckman Coulter,A54189, LS198-4-3	
CD45RA	1:100	Beckman Coulter,IM1834U, ALB11	
CD135	1:10	BD, 558996, Clone 4G8	
CD90	1:50	BioLegend, 328106, Clone 5E10	
Sytox blue	1:1000	Molecular probes, S34857	Dead cell stain
**Intracellular flow**			
CD34	1:100	BD,345804,8G12	
CD38	1:100	Biolegend, 356608,HB-7	
CD45RA	1:100	Biolegend, 304130,HI100	
53BP1	1:3000	Novus, NB100-304,polyclonal	

Alexa-488 Donkey anti-Rabbit	1:400	Molecular probes,A21206	
cPARP	1:100	Invitrogen, 44-699,polyclonal	
Zombie NIR	1:1000	Biolegend,423105	Fixable viability dye
**Western blot**			
P-ATM Ser51981	1:1000	EPITOMICS, EB1890Y	
P-DNA-PK Ser2056	1:500	ABCAM, ab18192	
P-CHEK2 Thr68	1:2000	Cell signaling, C13C1,	
P-CHEK2 Ser516	1:2000	Cell signaling, Antibody #2669, Polyclonal	
Actin	1:2000	Cell signaling, 8H10D10	

Supplementary Table 3. Gene expression datasets used for mRNA expression analysis

DataSet	Dataset title	Reference
GSE17054	Dysregulated gene expression networks in human acute myelogenous leukemia stem cells	(1)
GSE19599	Expression data for normal flow sorted hematopietic cell subpopulations	(2)
GSE11864	Effect of interferon-gamma on macrophage differentiation and response to Toll-like receptor ligands	(3)

E-MEXP-1242	Transcription profiling of human CD14+ CD16- and CD14low CD16+ monocytes from patients with Sjogrens syndrome and normal controls	(4)
GSE24759	Densely interconnected transcriptional circuits control cell states in human hematopoiesis	(5)
GSE42519	The Hematopoietic System - Myeloid arm	(6)

Supplementary Table 4. Gene sets used for mRNA expression analysis

**Gene Set**	**Systematic Name**	**Gene number**
KEGG Homologous Recombination	M11675	28
KEGG Non Homologous End Joining	M7857	14
DNA Damage Checkpoint	M7533	20
DNA Damage Response signal Transduction Resulting in Induction of Apoptosis	M13458	15

References

1. Majeti, R., Becker, M.W., Tian, Q., Lee, T.L., Yan, X., Liu, R., Chiang, J.H., Hood, L., Clarke, M.F. and Weissman, I.L. (2009) Dysregulated gene expression networks in human acute myelogenous leukemia stem cells. *Proc Natl Acad Sci U S A*, **106**, 3396-3401.
2. Andersson, A., Eden, P., Olofsson, T. and Fioretos, T. (2010) Gene expression signatures in childhood acute leukemias are largely unique and distinct from those of normal tissues and other malignancies. *BMC medical genomics*, **3**, 6.
3. Hu, X., Chung, A.Y., Wu, I., Foldi, J., Chen, J., Ji, J.D., Tateya, T., Kang, Y.J., Han, J., Gessler, M. *et al.* (2008) Integrated regulation of Toll-like receptor responses by Notch and interferon-gamma pathways. *Immunity*, **29**, 691-703.
4. Wildenberg, M.E., van Helden-Meeuwsen, C.G., van de Merwe, J.P., Drexhage, H.A. and Versnel, M.A. (2008) Systemic increase in type I interferon activity in Sjogren's syndrome: a putative role for plasmacytoid dendritic cells. *Eur J Immunol*, **38**, 2024-2033.
5. Novershtern, N., Subramanian, A., Lawton, L.N., Mak, R.H., Haining, W.N., McConkey, M.E., Habib, N., Yosef, N., Chang, C.Y., Shay, T. *et al.* (2011) Densely interconnected transcriptional circuits control cell states in human hematopoiesis. *Cell*, **144**, 296-309.
6. Rapin, N., Bagger, F.O., Jendholm, J., Mora-Jensen, H., Krogh, A., Kohlmann, A., Thiede, C., Borregaard, N., Bullinger, L., Winther, O. *et al.* (2014) Comparing cancer vs normal gene expression profiles identifies new disease entities and common transcriptional programs in AML patients. *Blood*, **123**, 894-904.

Original western blots

Related to figure S2A

.

1 2 3 4 5 6

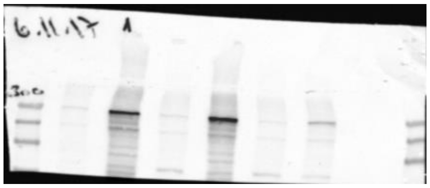

IB for pATM
(300KD)

IB for pATM
(300KD)

1 2 3 4 5 6

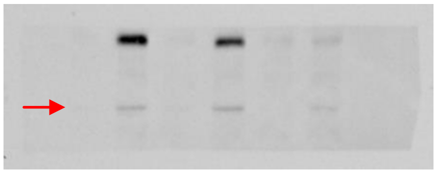

IB for pThr68
CHK2 (60KD)

IB for pATM
(300KD)

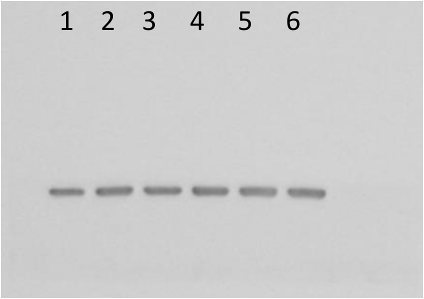

IB for Actin
(42KD)

IB for pATM
(300KD)

- Lanes 1,2 are non-relevant treatments

Related to figure S2B.

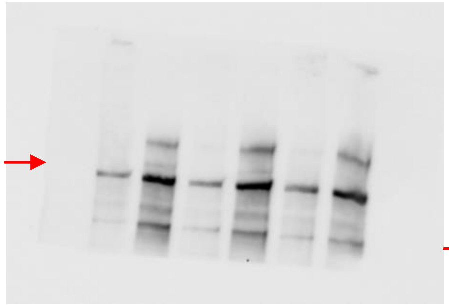

IB for pDNA-
PK (460KD)

IB for pATM
(300KD)

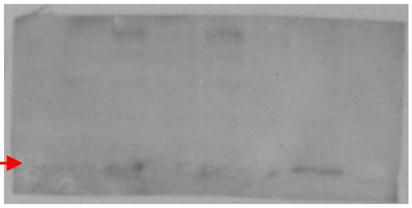

IB for pSer516
CHK2 (60KD)

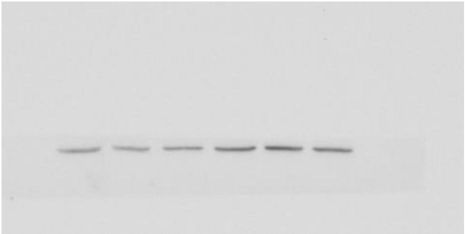

IB for Actin
(42KD)

IB for pATM
(300KD)

Related to figure S2C.

1 2 3 4 5 6 7 8

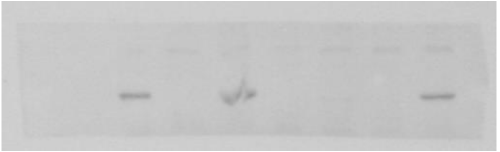

IB for pSer516
CHK2 (60KD)

- Lanes 7,8 are non-relevant treatments

1 2 3 4 5 6 7 8

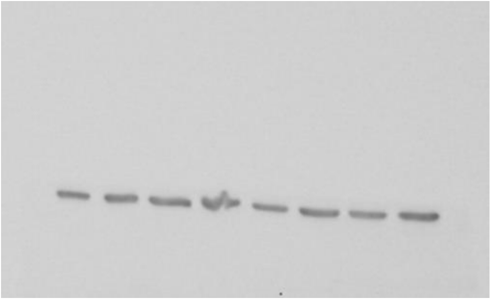

IB for Actin
(42KD)

IB for pATM
(300KD)
